# The role of resveratrol on skeletal muscle cell differentiation and myotube hypertrophy during glucose restriction

**DOI:** 10.1007/s11010-017-3236-1

**Published:** 2017-11-30

**Authors:** Hannah F. Dugdale, David C. Hughes, Robert Allan, Colleen S. Deane, Christopher R. Coxon, James P. Morton, Claire E. Stewart, Adam P. Sharples

**Affiliations:** 1 Institute for Science and Technology in Medicine (ISTM), School of Medicine, Keele University, The Guy Hilton Research Centre, Thornburrow Drive, Hartshill, Staffordshire ST4 7QB UK; 2 Stem Cells, Ageing and Molecular Physiology Research (SCAMP) Unit, Exercise Metabolism and Adaptation Research Group (EMARG), Research Institute for Sport and Exercise Sciences (RISES), Liverpool John Moores University, Liverpool, UK; 3 Department of Internal Medicine, Division of Endocrinology and Metabolism, Carver College of Medicine, University of Iowa, Iowa City, IA 52246 USA; 4 Centre for Applied Sport and Exercise Sciences, University of Central Lancashire, Preston, UK; 5 Department of Sport and Health Sciences, College of Life and Environmental Sciences, University of Exeter, Exeter, UK; 6 School of Pharmacy and Biomolecular Sciences, Liverpool John Moores University, Liverpool, UK

**Keywords:** SIRT1, Dietary restriction, Myoblasts, Hypertrophy, Atrophy, MYHC, P70S6K, AMPK, MYHC

## Abstract

Glucose restriction (GR) impairs muscle cell differentiation and evokes myotube atrophy. Resveratrol treatment in skeletal muscle cells improves inflammatory-induced reductions in skeletal muscle cell differentiation. We therefore hypothesised that resveratrol treatment would improve muscle cell differentiation and myotube hypertrophy in differentiating C2C12 myoblasts and mature myotubes during GR. Glucose restriction at 0.6 g/L (3.3 mM) blocked differentiation and myotube hypertrophy versus high-glucose (4.5 g/L or 25 mM) differentiation media (DM) conditions universally used for myoblast culture. Resveratrol (10 µM) treatment increased SIRT1 phosphorylation in DM conditions, yet did not improve differentiation when administered to differentiating myoblasts in GR conditions. Resveratrol did evoke increases in hypertrophy of mature myotubes under DM conditions with corresponding elevated *Igf-I* and *Myhc7* gene expression, coding for the ‘slow’ type I MYHC protein isoform. Inhibition of SIRT1 via EX-527 administration (100 nM) also reduced myotube diameter and area in DM conditions and resulted in lower gene expression of *Myhc* 1, 2 and 4 coding for ‘intermediate’ and ‘faster’ IIx, IIa and IIb protein isoforms, respectively. Resveratrol treatment did not appear to modulate phosphorylation of energy-sensing protein AMPK or protein translation initiator P70S6K. Importantly, in mature myotubes, resveratrol treatment was able to ameliorate reduced myotube growth in GR conditions over an acute 24-h period, but not over 48–72 h. Overall, resveratrol evoked myotube hypertrophy in DM conditions while favouring ‘slower’ *Myhc* gene expression and acutely ameliorated impaired myotube growth observed during glucose restriction.

## Introduction

Calorie Restriction (CR) promotes improvements in lifespan and healthspan in mammalian organisms due to chronic reductions in Insulin/Insulin-like-Growth-Factor-I (IGF-I) signalling, inflammation, DNA damage and oxidative stress (reviewed in [1]). However, reductions in IGF-I and downstream IGF-IR/protein kinase B (AKT)/mTOR/P70S6K negatively affect skeletal muscle mass via reductions in protein synthesis [2–6], muscle cell proliferation, differentiation and survival [7, 8]. In skeletal muscle, calorie restriction results in reduced IGF-I levels, a threefold reduction in Akt mRNA and 30–50% reduction in Akt protein activity, with corresponding increases in FoxO3a gene expression [9], leading to transcription of muscle-specific ubiquitin ligases/atrogenes MuRF-1 and MAFbx that ‘tag’ cytoskeletal and myofibrillar proteins for degradation in the proteasome [10–12]. Therefore, during CR a paradox exists where an attempt by the organism to improve longevity via reductions in IGF signalling are potentially at the expense of a loss in skeletal muscle regenerative capacity and mass (reviewed in [1]). As a consequence, using pharmacological or naturally derived agents that maintain muscle while calorie restricted would potentially be advantageous to improve health/lifespan while reducing the loss of muscle mass. Research into the activation of the Sirtuins (SIRT1-7), a group of protein deacetylases involved in the process of chromatin remodelling and gene regulation (see [13], by a naturally occurring polyphenol resveratrol (contained in the skin of red grapes), has provided some insights into the Sirtuins’ potential in both enabling improved healthspan and maintaining muscle cell regenerative function.

Indeed these insights suggest that reductions in SIRT1 activity in skeletal muscle occur following acute fasting in mice, with a corresponding increase in protein degradative MuRF-1 and MAFbx and reductions in muscle mass [14]. Our group has also shown that the induction of apoptosis and inhibition of differentiation in murine skeletal muscle cells by high-dose inflammatory cytokine, Tumour Necrosis Factor-alpha (TNF-α), was associated with increased SIRT1 mRNA levels, which when suppressed using silencing RNA resulted in exacerbated apoptosis [15, 16]. Importantly, the Sirtuin activator resveratrol was able to attenuate apoptosis and improve differentiation back towards baseline levels, suggesting that Sirtuin activation was important to survival and differentiation of skeletal muscle cells under a catabolic inflammatory stress. More recent studies have confirmed these findings, where resveratrol was also able to reverse impairments protein activity of Akt, mTOR, P70S6K and 4E-BP1 following TNF-α treatments in C2C12 cells [17]. It has also been demonstrated that resveratrol can reduce age-related ill health in *ad libitum* fed aged mice if administered from mid-age, albeit without impact on lifespan [18, 19], and can extend lifespan when mice are placed under nutrient stress from a high fat diet [20]. Therefore, resveratrol has the potential to reduce the impact of metabolic stress while helping maintain appropriate muscle cell function for skeletal muscle maintenance and regener ation.

Due to the requirement for adequate SIRT1 activation in muscle cell survival in vitro [16], an identified role for SIRT1 activation in regeneration/differentiation during inflammation in vitro and nutrient stress in vivo [20], we aimed to investigate whether Sirtuin activation via resveratrol treatment in skeletal muscle cells would attenuate loss of differentiation and myotube hypertrophy in both regenerating myoblasts and existing mature myotube cultures while modelling CR conditions in vitro. Reducing carbohydrate and glucose intake is a common approach when undertaking CR (reviewed in [21]). Therefore, glucose restriction (GR) has been used to model CR in vitro in cell types such as yeast [22], fibroblasts [23] and kidney cells [24]. In muscle cells, previous studies have used low glucose concentrations in vitro in an attempt to mimic circulatory glucose levels experienced during CR in vivo [25, 26]. However, the amount of glucose that muscle cells would actually be exposed to in the interstitial space during CR has not yet been modelled in vitro. The previously reported values for blood glucose during CR are 0.74 g/L (4.1 mM) in humans [27] and 1 g/L (5.6 mM) in murine models [28]. Glucose values in the muscle interstitial space have been reported to be 30% lower than those found in the blood of both rodents [29] and humans [30]. Therefore, applying the aforementioned 30% reduction would estimate glucose in the interstitial space at between 0.5 and 0.7 g/L (2.8 and 3.9 mM) for humans and rodents, respectively. Therefore, in the present study, we used 0.6 g/L of glucose in vitro to model interstitial glucose levels under CR in vivo. Both differentiating C2C12 myoblasts and mature myotubes were then investigated over time in GR conditions in the absence or presence of Sirtuin activator resveratrol and SIRT1 inhibitor, EX-527. These conditions were compared to conditions of 4.5 g/L (25 mM) high-glucose differentiation (DM) media that is universally and routinely used for both proliferating and differentiating skeletal muscle cells in vitro, with high-glucose DMEM used extensively in studies investigating the effect of resveratrol in C2C12 myoblasts [17, 31–34]. Overall, we hypothesised that (1) resveratrol treatment would help preserve differentiation and myotube hypertrophy in differentiating myoblasts under glucose restriction, and (2) resveratrol would improve myotube survival and prevent myotube atrophy in mature differentiated myotubes following glucose restriction (GR).

## Methods

### Cell culture

C2C12 murine myoblasts [35] between passage 8 and 10 were incubated in separate T75 flasks in a humidified environment (37 °C, 5% CO_2_) with growth media (GM) containing Dulbecco’s Modified Eagle Serum (DMEM) (D6429, Sigma-Aldrich, UK), 1% Penicillin Streptomycin (Pen-strep), 10% New born calf serum (NBCS) and 10% Foetal Bovine Serum (FBS) until 80% confluency was attained. Cells were trypsinized and cell counts were preformed using a haemocytometer in the presence of Trypan Blue dye. For studies in differentiating myoblasts, 6-well plates were pretreated with 0.2% porcine gelatin for 10 min at room temperature (RT) and 10 min in a humidified, 37 °C/5% CO_2_ environment. The excess gelatin was aspirated and cells were seeded at 8 × 10^4^ cells/ml in 2 ml of GM per well, these were then incubated for 24 h until 80% confluency was attained. Experiments were initiated by removing GM, washing twice with phosphate buffered saline (PBS) followed by the addition of low serum differentiation media for a total period of 7 days, containing: 8.3 g/L of DMEM (D5030, Sigma-Aldrich, UK), 0.584 g/L l-Glutamine, 3.7 g/L Sodium Bicarbonate, 0.11 g/L Sodium Pyruvate, 0.0159 g/L Phenol red, 2% horse serum and 1% Pen-strep and either 0.6 g/L or 3.3 mM (glucose restricted/GR) or 4.5 g/L (25 mM) high-glucose differentiation medium (DM) universally used for myoblast proliferation and differentiation and used extensively in previous studies assessing the role of resveratrol in C2C12 cells [17, 31–34], whilst also allowing relevant comparisons with the existing literature. The reduction in serum content causes C_2_C_12_ myoblasts to undergo spontaneous differentiation without requiring the addition of growth factors to initiate the process [35]. Time point zero was defined as an incubation of 30 min after transfer to DM and is denoted as 0 h (0 h). To assess the effect of resveratrol treatment and SIRT1 inhibition in myoblasts that were glucose restricted (GR) versus high-glucose differentiation media (DM), cells were incubated in either 0.6 g/L / 3.3 mM (GR) versus 4.5 g/L / 25 mM (DM) in the absence or presence of resveratrol (RES) at a concentration of 10 µM and SIRT1 inhibitor (EX-527) at 100 nM. Morphological analysis (myotube number, diameter and area), creatine kinase assays, and RNA extraction/ isolation for gene expression of genes associated with muscle cell differentiation (*myogenin)* and myotube maturation (*Myhc* 1, 2, 4, 7) were conducted at 0, 72 h and 7 days.

For studies in differentiated myotubes, myoblasts at passages 12–15 were washed in PBS and transferred into 2 ml of DM in 37 °C at 5% CO_2_ for 7 days in order to differentiate. Once myotubes had been formed over 7 days, cells were dosed in the below experimental conditions for a further 72 h (total time 10 days in culture) to assess the impact of resveratrol and EX-527 treatment during glucose restriction (GR) conditions in existing myotubes: GR differentiation media (0.6 g/L / 3.3 mM glucose alone), High-glucose differentiation media (DM) (4.5 g/L glucose alone), GR + Resveratrol (RES) (0.6 g/L glucose + 10 μM RES), DM + RES (4.5 g/L glucose + 10 μM RES), GR + EX-527 (0.6 g/L glucose + 100 nM EX-527) and DM + EX-527 (4.5 g/L glucose + 100 nM EX-527). Resveratrol/EX-527 was purchased from Merck Millipore (cat no 554325/566322, respectively, Nottingham, UK). Resveratrol was manufactured by Calbiochem (cat no CAS 501-36-0, San Diego, CA, USA). Resveratrol/EX-527 was reconstituted in DMSO and this stock was stored in -20 °C for up to three/six months, respectively, according to the manufacturer’s instructions. Morphological analysis of myotube number, diameter and area were performed 24, 48 and 72 h. For these experiments, the 0 h (0 h) baseline control condition was 7 days in DM to promote myotube formation, after which time cells were washed × 2 in PBS and then placed in fresh DM for 30 min containing (denoted DM 0 h). Protein activity of AMPK and P70S6K was analysed from protein lysates extracted at 0-, 15- and 30-min, 2- and 24-h time points after dosing occurred in myotubes in order to investigate energy sensing vs. protein synthetic/growth-associated cellular signalling following resveratrol and EX-527 administration in existing myotubes in GR versus DM glucose conditions. Gene expression for later differentiation and myotube maturation (*Mrf4, Myhc1, 2, 4, 7*) and genes associated with myotube hypertrophy (*Igf-I, Igf-Ir, Igf-II, Igf-IIr, Igfbp2, Mtor*), myotube atrophy (*Tnf-α, Tnfrsflb, Myostatin, Mur1f, Mafbx, Musa1, Fox01, 3, Nf-kb, p53*) as well as *Sirt1* gene expression were completed at 0, 24 and 72 h.

### Morphology to assess differentiation and myotube hypertrophy/atrophy

Myotube parameters including number, diameter and area were assessed using a live imaging light microscope (AF600 modular system, Leica, Germany) cell imaging system at ×10 or ×20 magnifications (see figure legends for details). Per experiment, each time point and experimental condition was imaged in duplicate with six images taken per well providing a total of 12 images per condition per time point per *n*. Experiments were then repeated *n* = 3. Automated mark and find on the Leica AF600 microscope allowed six images per well to be taken in the same position between conditions automatically, with the 6 locations chosen equally spread around the well. Analysis of myotube number, diameter and area was performed on the images using ImageJ software ((National Institutes of Health, USA) as previously defined in [36–38].

### Creatine kinase assay

Cells were extracted for total protein assays and CK (creatine kinase) activity (a biochemical marker of myoblast differentiation) at 0, 72 and 7 days in differentiating C2C12 myoblasts. Briefly, cells were washed twice in PBS and lysed in 250 µl well^−1^ of 0.05 M Tris/MES Triton lysis buffer (TMT: 50 mM Tris-MES, pH 7.8, 1% Triton X-100) and assayed using commercially available BCA^™^ (Pierce, Rockford, IL, U.S.A) and CK activity (Catachem Inc., Connecticut, N.E, USA) assay kits according to the manufacturer’s instructions. The enzymatic activity for CK was normalised to total protein content. CK and total protein were determined using a CLARIOstar^®^ plate reader (BMG labtech, Germany) at a wave length of 340 nm and 540–590 nm, respectively.

### RNA isolation and quantification

RNA isolation was performed using the TRIzol method, following the manufacturer’s instructions (Invitrogen, Life technologies, Carlsbad, CA). RNA purity and concentration were assessed using 1 μl of sample on a NanoDrop 2000c, UV–Vis (Ultraviolet–Visible spectroscopy) spectrophotometer (Thermo Fisher Scientific, MA, USA) using ODs of 230, 260 and 280 nm. A ratio of these OD value was calculated for each sample with all samples possessing 260/280 ratios of between 1.8 and 2.2 and therefore accepted as high enough quality RNA to enable downstream RT-PCR analysis.

### Primer design

Primer sequences (Table 1) were identified using Gene (NCBI, http://www.ncbi.nlm.nih.gov/gene) and designed using both web-based OligoPerfectTM Designer (Invitrogen, Carlsbad, CA, USA) and Primer-BLAST (NCBI, http://www.ncbi.nlm.nih.gov/tools/primer-blast), with the exception of IGF-I mature peptide mRNA primers that were used in [39]. Primers were purchased from Sigma (Suffolk, UK) without the requirement of further purification. Sequence homology (BLAST) searches ensured specificity to ensure the primers matched the sequence and therefore gene that they were designed for. Three or more GC bases in the last five bases at the 3′ end of the primer were avoided as stronger bonding of G and C bases can cause non-specific amplification. Primer sequences were designed to exclude hairpins, self-dimer and cross-dimers. All primers details can be found in Table 1.

**Table 1:** Primer details for gene expression analysis

Gene	Primer Sequence (5′-3′)	Reference number	Amplicon length (bp)
*MyoD*	F: CATTCCAACCCACAGAAC R: GGCGATAGAAGCTCCATA	NM_010866.2	125
*Mrf4 (Myf6)*	F: GGCTCTCCTTTGTATCCAGGG R: CGATCTGTGGGGGCAGATTT	NM_008657.2	194
*Myogenin*	F: CCAACTGAGATTGTCTGTC R: GGTGTTAGCCTTATGTGAAT	NM_031189.2	173
*Myhc1*	F: CGGTCGAAGTTGCATCCCTA R: TTCTGAGCCTCGATTCGCTC	NM_030679.1	149
*Myhc2*	F: GCGAAGAGTAAGGCTGTCCC R: GGCGCATGACCAAAGGTTTC	NM_001039545.2	76
*Myhc4*	F: AGGAGGCTGAGGAACAATCC R: TTCTCCTGTCACCTCTCAACA	NM_010855.3	192
*Myhc7*	F: TGTGCTACCCAGCTCCAAG R: CTGCTTCCACCTAAAGGGCTG	NM_080728.2	77
*Musa1*	F: CCTTGAGGCTCCCGGCAAAT R: ACTGCTCCACAAACCAATGGA	NM_001168297.1	189
*Sirt1*	F: ACAATTCCTCCACCTGAG R: GTAACTTCACAGCATCTTCAA	NM_019812.3	124
*Myostatin*	F: TACTCCAGAATAGAAGCCATAA R: GTAGCGTGATAATCGTCATC	NM_010834.3	194
*Igf-I*	F: GCTTGCTCACCTTTACCAGC R: TTGGGCATGTCAGTGTGG	NM_010512.5	280
*Igf-Ir*	F: TGCGGTGTCCAATAACTAC R: TGTTGATGGTGGTCTTCTC	NM_010513.2	110
*Igf-II*	F: GTACAATATCTGGCCCGCCC R: GTATGCAAACCGAACAGCGG	NM_010514.3	188
Igf-IIr	F: GGAACTCCTGAATTTGTAACT R: CTACCAGATAGCCACCATT	NM_010515.2	181
*Igfbp2*	F: AGTGCCATCTCTTCTACAA R: GCTCAGTGTTGGTCTCTT	NM_008342.3	197
*Mtor*	F: CACTCCACTATCCTGTTACCT R: GAGATCCTTGGCACACCT	NM_020009.2	190
*Murf1*	F: CCAAGGAGAATAGCCACCAG R: CGCTCTTCTTCTCGTCCAG	NM_001039048.2	84
*Mafbx*	F: GTCGCAGCCAAGAAGAGAA R: CGAGAAGTCCAGTCTGTTGAA	NM_026346.3	156
*FoxO1*	F: AGTGGATGGTGAAGAGCGTG R: GAAGGGACAGATTGTGGCGA	NM_019739.3	96
*FoxO3*	F: CGGACAAACGGCTCACTTT R: TCGGCTCTTGGTGTACTTG	NM_019740.2	272
Tnf-α	F: TCAACAACTACTCAGAAACAC R: AGAACTCAGGAATGGACAT	NM_013693.3	130
Tnfrsf1b	F: GTTGCTCTGTTATAGGATGGT R: TGCTGTCTGCTGTCTACT	NM_011610.3	114
*Nf-κβ*	F: ACACGAGGCTACAACTCTGC R: GGTACCCCCAGAGACCTCAT	NM_008689.2	164
*p53*	F: CATCCTGGCTGTAGGTAGCG R: GGCAGTCATCCAGTCTTCGG	NM_011640.3	109
*RpIIb* (a.k.a: *pol2rb*)	F: GGTCAGAAGGGAACTTGTGGTAT R: GCATCATTAAATGGAGTAGCGTC	NM_153798.2	197

### Gene expression by rt-qRT-PCR

Rt-qRT-PCR was carried out using Quantifast SYBR green RT-PCR kit (Qiagen, Manchester, UK) on a Rotor-Gene^®^ (Qiagen, Manchester, UK) supported by Rotor-Gene^®^ Q Software, version 2.1.0.9 (Qiagen, Manchester, UK). The rt-qRT-PCR cycles consisted of the following: 48 °C, 30 min (reverse transcription/ cDNA synthesis); 95 °C, 10 min (transcriptase inactivation and initial denaturation) followed by 40 cycles of 95 °C, 15 s (denaturation); 60 °C, 1 min (annealing and extension in 1 step). Disassociation melt-curve analysis was performed to reveal and therefore exclude non-specific amplification and primer dimer issues. All our gene products yielded a single melt peak/temperature suggesting that one product was amplified. Relative gene expression analysis was carried out using ΔΔC_t_ equation, otherwise known as the Livak method [40], this was to establish normalised expression ratios, where the relative expression was calculated as 2^−ΔΔCt^ and C_t_ represents the cycle threshold. RT-PCR efficiency was similar across RT-PCR runs and conditions (90.99 ± 2.28%, variation 2.48%). *Polr2ß* (a.k.a *Rp-IIb)* was also extremely stable between experimental conditions (mean C_t_ 15.62 ± 0.11) and therefore used as the reference gene in all RT-PCR assays and the pooled mean used in the ΔΔC_t_ calculations. All rt-qRT-PCR figures are presented as a relative gene expression in comparison to the 0 h cells incubated in DM glucose (4.5 g/L / 25 mM). This 0 h sample was used as a calibrator condition in the subsequent equations in order to compare expression values across glucose concentrations.

### SDS-PAGE and western blotting

Cells were lysed in 300 µl lysis buffer per well of a 6-well plate, including phosphatase inhibitors (10 mM TrisHCL, 5 mM EDTA, 50 mM Sodium Chloride, 30 mM Sodium Pyrophosphate, 50 mM Sodium Fluoride, 100 μM Sodium Orthovanadate, 1 mM PMSF and 1% Triton X-100.) supplemented with protease inhibitor tablets as per the manufacturer’s instructions (Roche, Switzerland). Following analysis of total protein via BCA assay as above, 30 µg of protein was reconstituted in 1:5 dilution with 5X Laemmli buffer (3 ml 1M TRIS–HCl (pH 6.8.), 1 g Sodium dodecyl sulphate, 5 ml glycerol, 1 ml DH_2_O and 25 mg Bromophenol blue). Ten percent resolving SDS-Polyacrylamide (SDS-Page) gels (4 ml 30% acrylamide 1% BIS solution, 3.4 ml DH_2_O, 2.5 ml 1.5M Tris Base, 100 μl 10% SDS, 50 μl 10% APS and 5 μl TEMED) were poured, and a layer of butanol was then syringed across the top of the resolving solution and cast for 30 min. Following butanol removal, a 5% stacking gel solution (1.7 ml 30% acrylamide 1% BIS solution, 5.7 ml DH_2_O, 2.5 ml 0.5M Tris Base, 100 μl 10% SDS, 50 μl 10% APS and 10 μl TEMED) was poured and a lane comb inserted. These gels were run at 200 V in a Mini- PROTEAN^®^ Tetra vertical electrophoresis cell (Bio-Rad Laboratories, Inc. CA, USA) until the bromophenol blue dye line reached the bottom of the gel (approximately 1 h). The protein was then transferred for 30 min at 200 V onto a nitrocellulose membrane within a semi-dry Trans-blot^®^ Turbo^™^ Blotting system cassette (Bio-Rad Laboratories, Inc. CA, USA) using transfer buffer (20 ml 10 × Tris Glycine, 40 ml methanol and 140 ml DH_2_O). The nitrocellulose membrane was prepared for detection of specific phosphorylated and total proteins using a Pierce^™^ Fast Western Kit, Supersignal West Pico (Rabbit) (Thermofisher scientific, MA, USA) as per the manufacturer’s instructions including timings for incubation, with the following primary antibodies (all raised in rabbit) purchased from Cell Signalling Technology, MA, USA unless otherwise stated: Phospho AMPK 1:1000 (cat no: #2535), total AMPK 1:1000 (#2532), phospho P70S6K 1:1000 (#9205), total P70S6K 1:1000 (#9202), phospho SIRT1 1:2000 (#2314L) and total SIRT 1:2000 (Millipore, Watford, UK cat no: #07-131). Incubation with the secondary rabbit HRP antibody, was also provided in the above Pierce^™^ Fast Western Kit and applied as per the manufacturer’s instructions. Enhanced chemilluminescence (ECL) detection reagents as provided by the Pierce^™^ Fast Western Kit described above were used in a 1:1 dilution and incubated over the membrane for 5 min. The membrane was then placed in the Chemidoc^™^ MP System (Bio-Rad Laboratories, Inc. CA, USA.) where the band images were detected by densitometry in which the first image was taken at 5 s and intervals of 30.5 s thereafter. Following imaging, the band volumes were detected using Image lab^™^ (Bio-Rad Laboratories, Inc. CA, USA.). The bands for the phosphorylated protein were relativised to its own total protein counterpart before determining changes between experimental groups. GAPDH was detected on all membranes prior to further detection. To establish whether loading of protein was comparable, we determined the volume values for GAPDH (GAPDH concentration 1:4000, Cell Signalling Technology, MA, USA cat no: #5174). In the unlikely event that GAPDH was significantly different between conditions, the samples were also relativised to the GAPDH loading control.

### Statistical analysis

All data analyses were carried out using Minitab^®^ 17 (Minitab Ltd, Coventry U.K). Outliers were removed using Grubbs outlier test. All data were parametric, assessed using the Anderson–Darling test for normality. For statistical analysis of the dependent variables investigated in differentiating myoblasts, a general linear model (2 × 2 × 3) for time (72 h, 7 days), glucose concentration (GR, DM) and resveratrol/EX-527 treatment (DM, RES, EX-527) where carried out for morphological analysis of myotube number, diameter and area. For CK activity and gene expression, a general linear model (4 × 2 × 3) for time (0, 24, 72 h, 7 days), glucose concentration (GR, DM) and resveratrol/EX-527 treatment (DM, RES, EX-527) was conducted. For statistical analysis of the dependent variables investigated in differentiated myotubes (already differentiated for 7 days prior to treatments), a general linear model (2 × 2 × 3) for time (0, 72 h), glucose concentration (GR, DM) and resveratrol/EX-527 treatment (DM, RES, EX-527) where carried out for morphological analysis of myotube number, area and diameter for the 72-h data. Morphological analysis for the additional 24-h and 48-h data was also performed subsequently using a general linear model (2 × 2 × 3) for time (0, 24 and 48 h), glucose concentration (GR, DM) and resveratrol/EX-527 treatment (DM, RES, EX-527). Gene expression data for both the 72-h and 24-h data were performed using a general linear model (2 × 2 × 3) for time (0, 72 h or 0, 24 h), glucose concentration (GR, DM) and resveratrol/EX-527 treatment (DM, RES, EX-527). A general linear model for glucose (LOW, NOR) and resveratrol/EX-527 treatment (DM, RES, EX-527) was performed for phosphorylated protein activity. Relevant post hoc corrections tests were performed to identify significant comparisons. Statistical significance was at the level of *p* ≤ 0.05.

## Results

### The effect of resveratrol and glucose restriction on myoblast differentiation and myotube hypertrophy

**Fig. 1:**
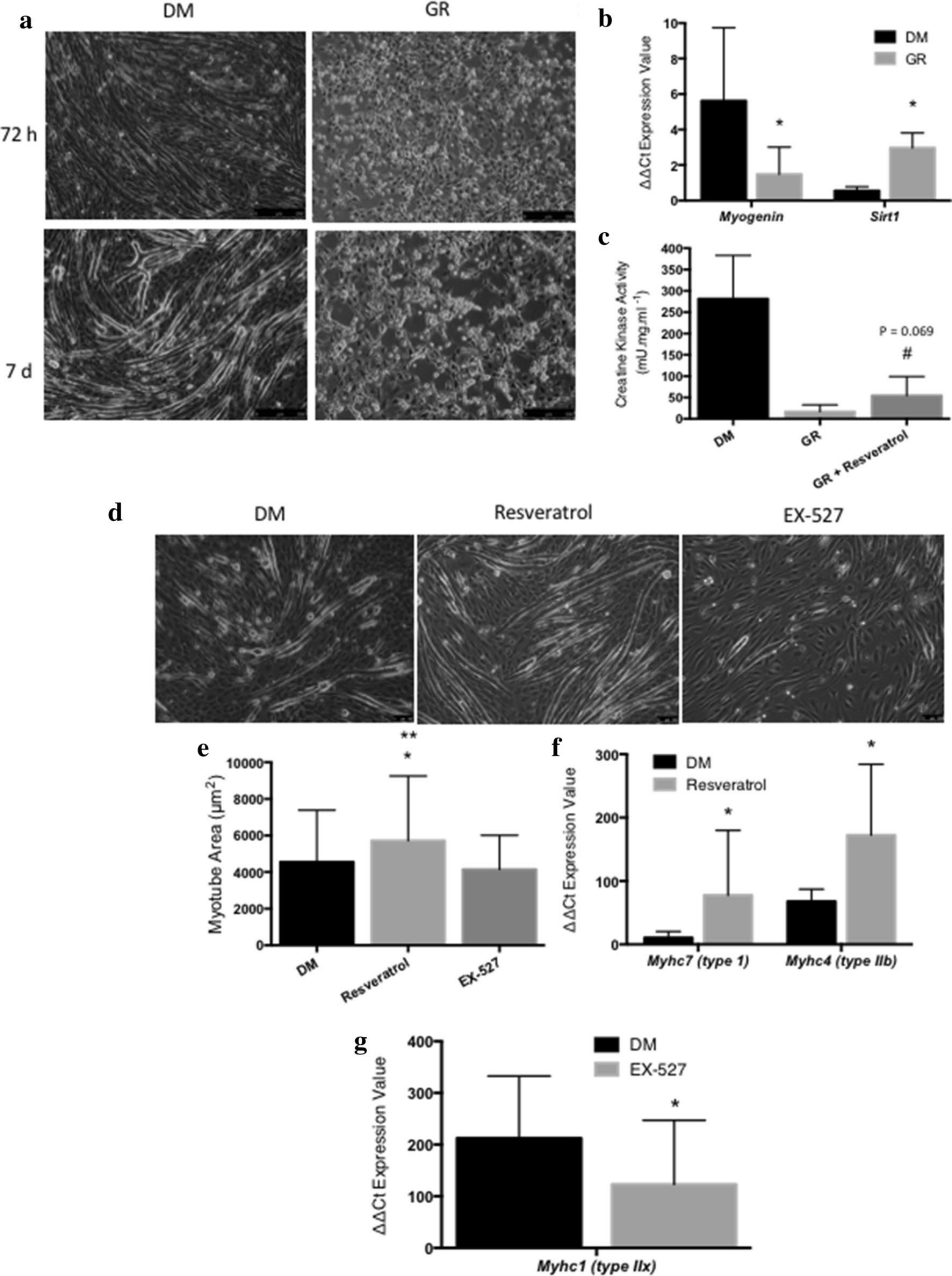
**a** Glucose Restriction (GR) blocked differentiation in differentiating myoblasts with significant reductions in; **b** *myogenin* and increases in *Sirt1* gene expression. Resveratrol improved, **c** CK activity in GR conditions # (albeit non-significantly *p* = 0.069), without improvements in differentiation. Resveratrol also improved, **d, e** myotube area when glucose was more readily available in DM (differentiation media control) conditions at 7 days post induction of differentiation. This was associated with increases in **f** *Myhc*7 and *Myhc*4 coding for the slow type I and fast IIb MYHC protein isoforms. Further, EX-527 administration in DM conditions resulted in reductions in **g** *Myhc1* coding for IIx MYHC protein isoform with this condition displaying the smallest mean myotube size versus all other conditions. *Significantly different (*p* ≤ 0.05) versus DM conditions. **Significantly, (*p* ≤ 0.05) different versus EX-527 conditions. All experiments are at least *n* = 3 in duplicate

SIRT1 is a histone deacetylase and phosphorylation of SIRT1 is required to enable increases in deacetylase activity [41]. Therefore, in order to first assess the optimal dose of resveratrol and EX-527 in C2C12 cells, SIRT1 phosphorylation was assessed in DM control glucose conditions. Indeed, SIRT1 phosphorylation increased on average by 15-fold versus DM control when relativised to total SIRT1 after 24-h administration of resveratrol. The higher dose of 15 µM resveratrol showed no additional increase versus 10 µM (where 5 µM resulted in no significant increase in SIRT1 phosphorylation) and higher doses of 30 and 60 µM of resveratrol were cytotoxic and evoked cell death (data not shown) as suggested in previous independent studies using these doses in C2C12 cells [33]. Following the addition of 100 nM EX-527, we observed an average decrease of 71% in SIRT1 phosphorylation following 24 h in comparison to the DM control at 15 min, compared to only a 50% reduction after 24 h in SIRT1 phosphorylation without EX-527 administration. Following these initial experiments, we next sought to assess the role of glucose restriction alone on myoblast differentiation. Glucose restriction completely blocked differentiation in myoblasts demonstrated by no myotube formation in these conditions (Fig. 1a). This corresponded with a significant reduction in myogenin gene expression at 72 h versus DM conditions (GR; 1.48 ± 1.53 vs. DM; 5.62 ± 4.12, *p* = 0.047, Fig. 1b). This was associated with significant increases in *Sirt1* transcript expression in GR conditions at 72 h (GR vs. DM: 2.97 ± 0.84 vs. 0.55 ± 0.23, *p* ≤ 0.05, Fig. 1b), a similar finding to that demonstrated previously upon blocking differentiation in the presence of the inflammatory cytokine TNF-α [16]. Resveratrol (10 μM) was also unable to improve *myogenin* expression (data not presented) or prevent the block on morphological differentiation in GR conditions. Despite this, resveratrol improved biochemical marker of differentiation, creatine kinase (CK), in GR conditions at 7 days (GR: 16.82 ± 15.95 vs. GR RES 54.4 ± 44.7 mU mg ml^−1^, *p* = 0.069; Fig. 1c), albeit not quite attaining statistical significance. However, changes in CK activity were also temporally delayed, where increases are usually observed 48–96 h post serum withdrawal in normal glucose conditions [42–44]. Further, this non-significant increase in CK activity was without improvement in morphological differentiation, where CK levels did not approach those observed in DM glucose conditions alone (281.5 ± 101.5 mU mg ml^−1^). Further, resveratrol was unable to improve myotube number or diameter in DM conditions, however, did significantly improve myotube area after 7 days of differentiation versus DM alone and versus EX527 administration (DM; 4551 ± 2836 μm^2^ vs. DM RES; 5718 ± 3532 μm^2^, *p* = 0.044, vs. DM EX 527; 4142 ± 1873 μm^2^, *p* = 0.021, Fig. 1d, e), suggesting an increase in myotube length versus width. This was associated with a corresponding increase in *Myhc*7 and *Myhc*4 coding for the slow type I and fast IIb MYHC protein isoforms, respectively, in resveratrol versus DM alone conditions (Myhc7: DM vs. DM RES: 10.53 ± 9.84 vs. 77.5 ± 102.4, *p* = 0.002, *Myhc4*: DM vs. DM RES: 67.9 ± 19.2 vs. 172.4 ± 111.8, *p* < 0.001, Fig. 1f). Furthermore, EX-527 administration resulted in reductions in *Myhc1* coding for intermediate IIx MYHC protein isoform (DM vs. DM EX527: 212.4 ± 120.4 vs. 123 ± 124, *p* = 0.008, Fig. 1g), with this condition displaying the smallest mean myotube size (4142 ± 1873 μm^2^, Fig. 1e). Overall, resveratrol treatment in normal glucose conditions evoked higher gene expression associated with a slow and fast fibre formation and EX-527 administration resulted in lower gene expression associated with intermediate fibre formation. However, resveratrol treatment was unable to prevent the complete block on differentiation, despite improved CK activity, in glucose-restricted conditions.

### Resveratrol was unable to prevent reduced myotube growth in glucose restriction conditions yet improved myotube hypertrophy when glucose was available

**Fig. 2:**
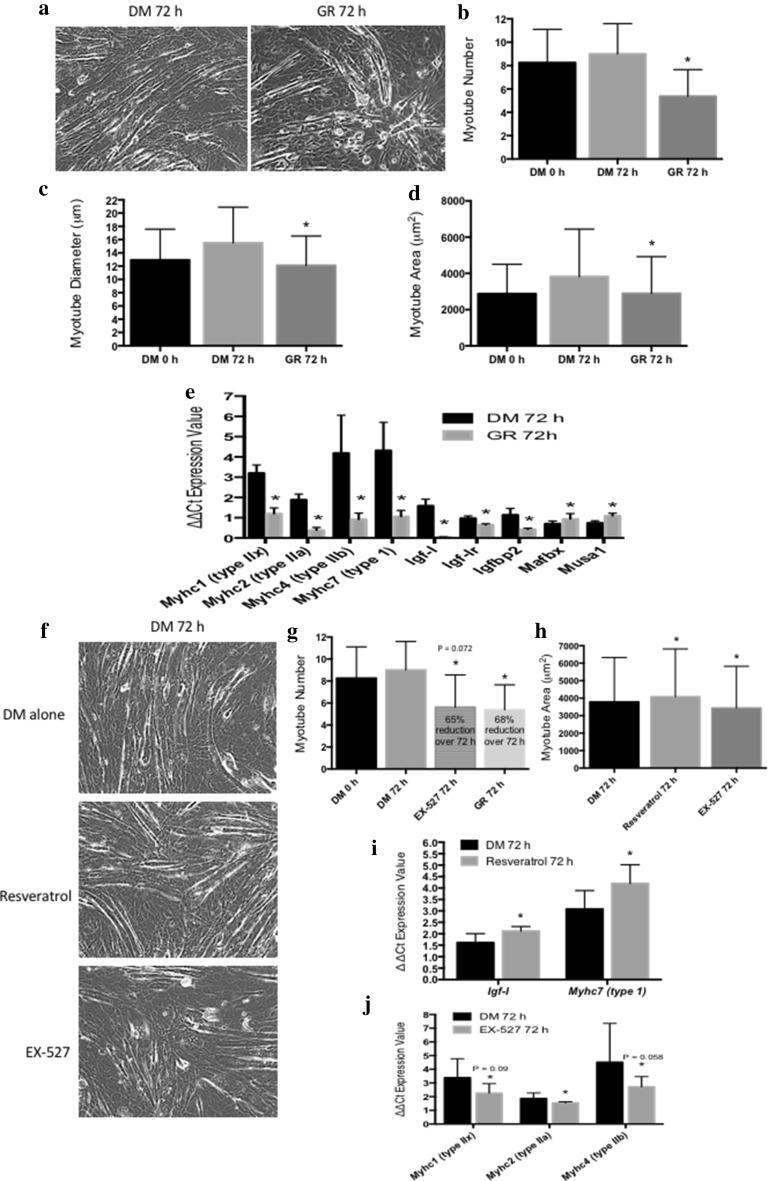
Loss of myotubes and reduced myotube growth occurred when existing myotubes were placed in Glucose Restriction (GR) conditions for 72 h, demonstrated **a** morphologically and via reductions in **b** myotube number, **c** myotube diameter and **d** myotube area. This corresponded with reductions in gene expression of myotube maturation genes **e** *Myhc1, 2, 4, 7*, reductions in genes associated with myofibre hypertrophy *Igf-I, Igf-Ir* and *Igfbp2* and increased expression of genes associated with myotube atrophy/protein degradation, *Mafbx* and *Musa1*. Resveratrol was unable to improve myotube survival or reduced myotube growth in GR conditions at 72 h however, resveratrol evoked increases in **f, h** myotube area at 72 h when glucose was more readily available (DM/differentiation media control conditions). This was associated with increases in **i** Igf-I and *Myhc7* that codes for the slow type I *Myhc* protein isoform. EX-527 treatment also reduced **g** myotube number and **h** myotube area. This was associated with reductions in gene expression of **j** *Myhc* 1, 2 and 4 coding for intermediate and IIx, IIa and IIb MYHC protein isoforms, respectively. It is worth noting EX-527 administration evoked a similar 65–68% loss of myotubes experienced in GR conditions alone. *Significantly different (*p* ≤ 0.05) versus DM 72-h conditions. All experiments are at least *n* = 3 in duplicate

Since resveratrol induced changes in *Myhc* gene expression were at later time points of differentiation in myoblasts, we decided to investigate the role of resveratrol and EX-527 treatment in both DM and glucose restriction (GR) conditions in existing myotubes (already matured for 7 days). Firstly, we confirmed that GR alone evoked a considerable reduction in myotube number (DM 9 ± 2.85 vs. GR 5.38 ± 2.28, *p* ≤ 0.05, Fig. 2a, b), and reduced myotube growth demonstrated by reductions in myotube diameter (DM 15.56 ± 5.38 vs. GR 12.1 ± 4.453 μm, *p* ≤ 0.05, Fig. 2a, c) and area (DM 3820 ± 2625 vs. GR 2897 ± 2024 μm^2^, *p* ≤ 0.05, Fig. 2 a, d) in existing myotubes after 72-h glucose restriction. These morphological observations were confirmed in GR alone versus DM conditions, where lower gene expression of myotube maturation genes *Myhc1, 2, 4, 7* (*Myhc1*: DM 3.2 ± 0.4 vs. GR 1.2 ± 0.29; *Myhc2*: 1.86 ± 0.28 vs. GR 0.39 ± 0.13; *Myhc4*: DM 4.19 ± 1.87 vs. GR 0.92 ± 0.31; *Myhc7*: DM 4.32 ± 1.39 vs. GR 1.06 ± 0.3, all *p* ≤ 0.05, Fig. 2e), and genes associated with myotube hypertrophy were observed (*Igf-I; Igf-Ir and Igfbp2 (Igf-I: DM* 1.59 ± 0.32 vs. GR 0.05 ± 0.01; *Igf-Ir*: DM 0.97 ± 0.1 vs. GR 0.64 ± 0.06; *Igfbp2*: DM 1.14 ± 0.32 vs. GR 0.43 ± 0.05, all *p* ≤ 0.05, Fig. 2e). This also was associated with higher expression of genes associated with protein degradation such as *Mafbx and Musa1 (Mafbx: DM* 0.7 ± 0.14 vs. GR 0.93 ± 0.27; *Musa1*: DM 0.74 ± 0.1 vs. GR 1.09 ± 0.13, all *p* ≤ 0.05, Fig. 2e*)*. No changes were observed in gene expression of *Igf-II, Igf-IIr, Mtor, Tnf-α, Tnfrsflb, Myostatin, Murf, Fox01, Fox03, Nf-kb, p53 or Sirt1* (data not shown). Resveratrol did not prevent the reductions in myotube growth in GR conditions, however importantly, resveratrol administration was able to promote significant increases in myotube hypertrophy at 72 h when added to existing myotubes after 7 days of differentiation in DM conditions (DM; 3785 ± 2542 vs. DM RES 4088 ± 2728 μm^2^, *p* = 0.05, Fig. 2f, h). This corresponded with significant increases in the expression of genes associated with myotube hypertrophy such as *Igf-I (*DM; 1.61 ± 0.40 vs. DM RES; 2.12 ± 0.20, *p* = 0.033; Fig. 2i) *(*with no change in *Igf-Ir, IGF-II, Igf-IIr, or Mtor*) and *Myhc7*, that codes for the slow type I *Myhc* protein isoform (DM; 3.08 ± 0.81 vs. DM RES; 4.20 ± 0.82, *p* = 0.002, Fig. 2i). Resveratrol however, did not affect expression of genes associated with myotube atrophy/protein degradation (*Tnf-α, Tnfrsf1b, Myostatin, Murf, Mafbx, Musa1, Fox-01, 3, Nf-kb, p53*, data not shown) in this condition. Alternatively, EX-527 treatment (100 nM) also reduced myotube number, an observation that approached significance (DM 9.00 ± 2.59 vs. DM EX-527 5.62 ± 2.94, *p* = 0.073, Fig. 2g), and myotube area at 72 h (DM 3785 ± 2542 vs. DM EX-527; 3433 ± 2394 μM^2^, *p* = 0.026, Fig. 2h). Indeed, the loss of myotube number with EX-527 administration at 72 h in high-glucose conditions was similar to the loss observed in GR conditions alone, confirmed by no significant difference between these conditions (DM EX-527 5.62 ± 2.94 vs. GR 5.38 ± 2.28, *p* = N.S.) and both GR and DM EX-527 conditions exhibited a similar ~ 65% reduction in myotubes vs. 0 h controls (0 h, 8.25 ± 2.85 vs. DM EX-527 5.62 ± 2.94 (65% reduction) vs. GR 5.38 ± 2.28 (68% reduction), both comparisons *p* ≤ 0.001, Fig. 2g). In these conditions where EX-527 was administered yet glucose was more readily available, there were also reductions in gene expression of *Myhc* 1, 2 and 4 coding for intermediate/faster IIx, IIa and IIb protein isoforms, respectively, (*Myhc1*; DM 3.39 ± 1.38 vs. DM EX-527 2.26 ± 0.69, p = 0.09: *Myhc2*; DM 1.86 ± 0.41 vs. DM EX-527 1.55 ± 0.08, *p* = 0.034: *Myhc4*; DM 4.52 ± 2.83 vs. DM EX-527 2.72 ± 0.74, *p* = 0.058 (Fig. 2i). Overall, these data suggest that where glucose was readily available, resveratrol and EX-527 promoted myotube hypertrophy and reduced growth, respectively. Furthermore, resveratrol administration regulated *Myhc* genes towards a ‘slower’ fibre-type expression profile vs. a reduced intermediate/faster expression profile in the presence of EX-527. Finally, in these conditions, EX-527 administration evoked a similar 65% loss of myotubes, also experienced in GR conditions alone.

### Resveratrol administration acutely negated reduced myotube growth during glucose restriction

**Fig. 3:**
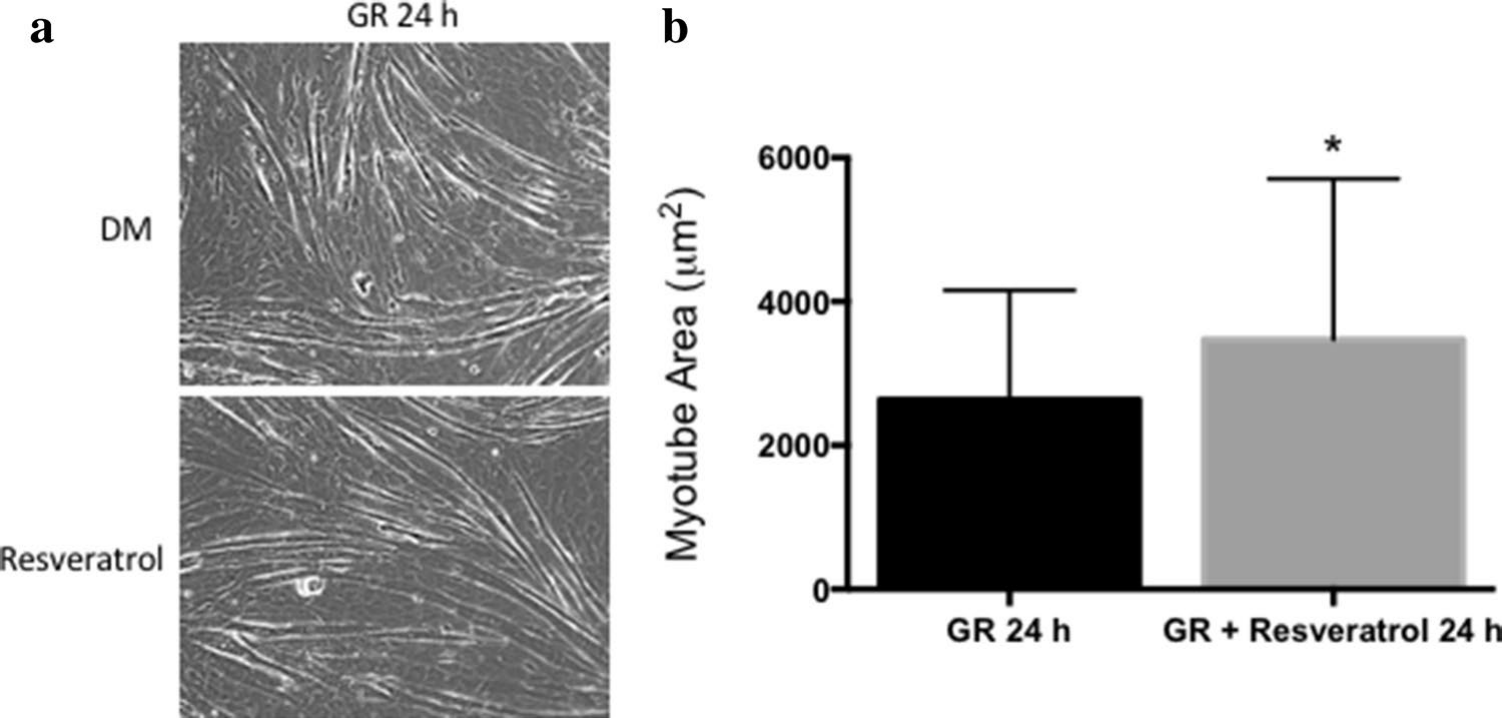
Resveratrol was able to improve **a, b** myotube area for a period of 24 h after dosing in Glucose Restriction (GR) conditions. *Significantly different (*p* ≤ 0.05) versus GR conditions alone. All experiments are at least *n* = 3 in duplicate

**Fig. 4:**
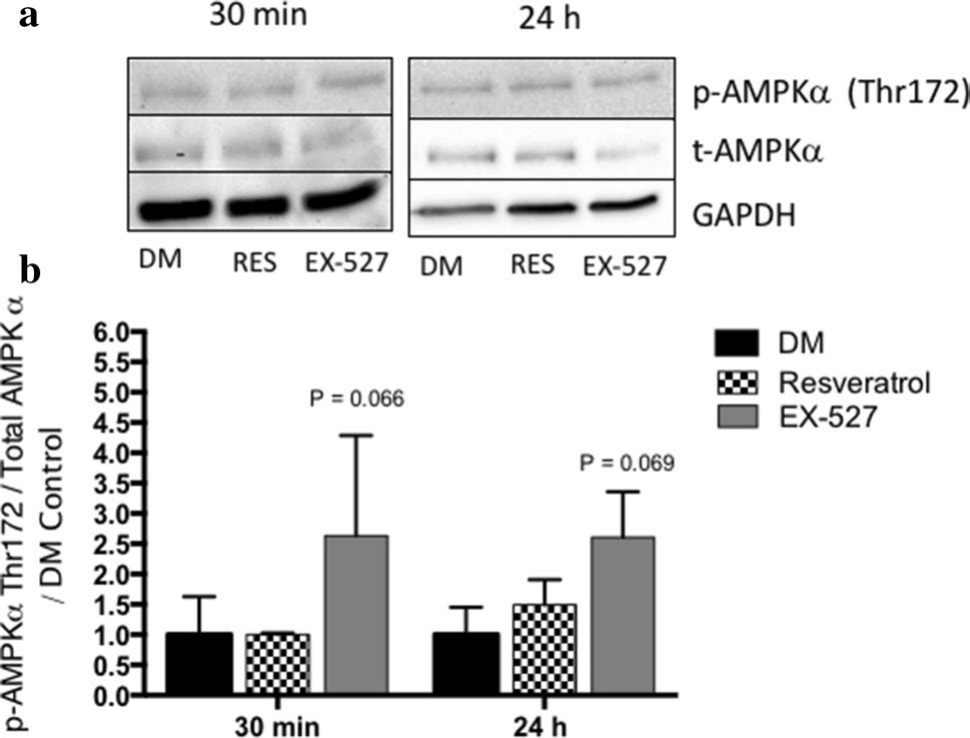
Resveratrol treatment did not alter the activity of energy-dependent sensing protein AMPK or protein translation initiator P70s6K in Glucose Restriction (GR) conditions over 24 h. However, EX-527 treatment on average increased **a, b** (approached significance) AMPK activity at 30 min and 24 h in DM (differentiation media control) conditions (level of significance depicted on Fig. 2b). All experiments are at least *n* = 3

Importantly, resveratrol treatment in existing myotubes under GR conditions for 24 h was also able to improve myotube hypertrophy (Fig. 3a) demonstrated by significant improvements in myotube area (GR 2635.30 ± 1524.10 vs. GR RES: 3474.00 ± 2235.00 μM^2^, *p* < 0.001, Fig. 3b). However, this was without changes in *Myhc* gene expression profiles as observed above in DM conditions (data not shown). Despite this improvement after 24 h, resveratrol was unable to improve myotube size in GR conditions at 72 h when administered to existing myotubes (data not shown). Therefore, a single dose of resveratrol had a role in acutely (for 24 h) negating the effect of reduced myotube growth in vitro when modelling interstitial glucose levels of CR in vivo. Finally, because of the improvements in myotube size over an acute 24-h period with resveratrol treatment in GR conditions yet without alterations in gene expression of myosin heavy chains, alterations in more acutely and transiently regulated cell signalling activity of energy-sensing protein (AMPK) and protein translation initiator (P70S6K) were assessed at 15, 30 min, 2 and 24 h in GR and DM conditions in the absence and presence of resveratrol and EX-527. Indeed, it has previously been shown that AMPK increases following starvation and suppresses activity of protein synthetic signalling (P70S6K) [24]. Resveratrol did not alter the activity of energy-dependent sensing protein AMPK or protein translation initiator P70S6K in glucose-restricted conditions (data not shown). However, EX-527 administration on average increased (approaching significance) AMPK phosphorylation at 30 min in DM glucose conditions (30 min; DM 1.00 ± 0.63 vs. DM EX-527 2.63 ± 1.66, *p* = 0.066, 2 h DM vs. DM EX-527: 1.00 ± 0.36 vs. 2.24 ± 1.45, *p* = 0.086) and 24 h (DM vs. DM EX-527: 1.00 ± 0.43 vs. 2.61 ± 0.75, *p* = 0.069; Fig. 4a, b). Increases in AMPK with EX-527 in DM glucose conditions also led to a non-significant reduction in protein activity of P70S6K at 30 min versus DM alone (DM vs. DM EX-527: 1.00 ± 0.56 vs. 0.76 ± 0.15, *p* = N.S). However, similar non-significant reductions were observed with resveratrol treatment. Overall, these signalling data perhaps suggest that adequate Sirtuin activity is required to enable normal AMPK signalling with normal glucose availability; however, resveratrol did not increase P70S6K signalling in either GR or DM conditions.

## Summary/discussion

In the present study, we hypothesised that resveratrol treatment in skeletal muscle cells would attenuate loss of differentiation and reduced myotube size in both regenerating myoblasts and existing mature myotubes, respectively, during glucose restriction. This hypothesis however was partly rejected because, despite confirming increased SIRT1 phosphorylation with resveratrol administration in DM conditions, we were unable to prevent the block on differentiation in myoblasts caused by GR conditions in the presence of resveratrol (10 µM). Importantly however, resveratrol was able to evoke increases in myotube area of differentiating myoblasts under DM glucose conditions with corresponding increases in both *Myhc7* and 4 coding for the both slow type I and fast IIb *Myhc* protein isoforms, respectively. Our original hypothesis however could be partly accepted in experiments undertaken at more acute time points in mature myotubes. Resveratrol treatment in mature myotubes under GR conditions was able to improve myotube hypertrophy over an acute 24-h period, but not over 48–72 h. However, this observation was without changes in *Myhc* gene expression profiles as previously observed in DM conditions where glucose was available with resveratrol treatment. This suggested that a single dose of resveratrol played a role in acutely (for 24 h) negating the effect of reduced myotube growth when modelling in vitro interstitial glucose-restricted levels during CR in vivo. Despite this, resveratrol did not appear to modulate phosphorylation of energy-sensing protein AMPK or protein translation initiator P70S6K in these myotubes. EX-527 (SIRT1 inhibitor) administration did however, increase average AMPK phosphorylation in both GR and DM (that approached significance) glucose conditions with a corresponding average, yet non-significant suppression of P70S6K in DM glucose conditions. Finally, in myotubes at later time points (72 h) where glucose was readily available, resveratrol treatment promoted myotube hypertrophy and regulated *Myhc7* genes towards ‘slower’ fibre type expression profiles, with SIRT1 inhibition via EX-527 administration also causing a reduction in *Myhc* 1, 2 and 4 coding for intermediate and faster IIx, IIa and IIb protein isoforms, respectively.

Previous studies have suggested that total MYHC protein and myotube size (diameter) are increased when glucose is readily available in the presence of 20 µM resveratrol [45]. Other studies have suggested that overexpression of SIRT1 in mouse and human skeletal muscle cells, impairs differentiation and myosin heavy chain production, however, if SIRT1 is decreased then muscle cells differentiate prematurely [46, 47]. Furthermore, an increase in proliferation, inhibition of p21cip and p27kip (cyclin-dependent kinase inhibitors important for cell cycle exit in G1 in order to enable myoblast fusion) caused a reduction in differentiation following SIRT1 overexpression in rat myoblasts [48]. These studies would therefore somewhat disagree with the present investigation. However, more recently it has been suggested that a resveratrol concentration of 10 µM (the same as the present study) increased the percentage of cells in the G1 phase cell cycle exit required for myoblast differentiation [33]. The present study consolidated the role of increased SIRT1 phosphorylation in differentiating myoblasts via resveratrol treatment when glucose is readily available via the regulation of increases in *Myhc* gene expression (slow and fast) and increased myotube size, together with increases in slow Myhc gene expression in myotubes. Furthermore, SIRT1 inhibition via EX-527 administration resulted in reductions in intermediate (in myoblasts) and intermediate/fast (in myotubes) *Myhc* gene expression, suggesting that normal SIRT1 activity is perhaps required to maintain adequate intermediate to fast *Myhc* gene expression. Indeed, previous studies have suggested that transgenic overexpression of SIRT1 evokes a slow phenotype in mice [49]. Due to this interesting finding, future studies may wish to investigate the role of SIRT1 activation/inhibition on Nuclear factor of activated T-cells (NFAT) activity in skeletal muscle cells. As this transcription factor regulates the activation of muscle fibre genes associated with the characterization of ‘slow’ and ‘fast’ myofibres [50], and is known to be transcriptionally suppressed by SIRT1 [51]. Finally, if extrapolating to the potential use of resveratrol in elderly populations in vivo to attenuate age-related muscle loss (sarcopenia). Sarcopenia is characterised by a more predominant loss of type II fibres [52] and therefore is a contributing factor to reductions in maximum strength with age (reviewed in [53]). Data herein suggest resveratrol treatment, although resulting in increased myofibre size, these fibres may be slower in fibre composition which may not be advantageous in terms of force production, but perhaps may contribute to improvements in metabolic health in vivo [54].

As discussed above, although resveratrol treatment was unable to restore blocked differentiation in myoblasts under GR conditions, resveratrol was able to improve myotube diameter and area in GR compared with GR alone conditions in mature myotubes over an acute period of 24 h. However, this finding did not continue over the remaining time course (48–72 h) where myotubes were then lost (reduced number) under GR conditions even in the presence of the resveratrol. The improvement in myotube size at 24 h with resveratrol in GR conditions were not driven by changes in Myhc gene expression, as was the case in control conditions where glucose was more readily available. From these findings, we can infer that the reduced myotube growth experienced under GR conditions may be attenuated following a single dose of resveratrol over a 24-h time period. However, repeated doses may be required to maintain myotube hypertrophy in vitro over a longer time period under GR conditions. Yet, this approach in vivo requires further investigation, due to altered pharmacokinetics and high absorption, yet low bioavailability through the gut after oral administration [55]. Due to no changes in Myhc gene expression at an acute time point of 24 h in myotubes under GR conditions in the presence of resveratrol, yet an increase in myotube size was observed; we investigated more acute and transient mechanisms of intracellular signalling of protein synthetic translation initiator P70S6K at 15, 30 min, 2 and 24 h after dosing. Furthermore, because glucose restriction in skeletal muscle cells has been shown to increase activity of AMPK [47] and can suppress growth-related signalling of P70S6K via TSC2 inhibition of mTOR [24], we also investigated AMPK signalling at the same time points. Despite this, there were no changes in phosphorylation of AMPK or P70S6K in GR or DM conditions with resveratrol versus relevant control conditions. However, we did observe a trend towards increased AMPK following EX-527 administration, which approached significance in DM conditions together with average but non-significant reduction in P70S6K, where in this condition there was a corresponding suppression of intermediate/fast *Myhc* gene expression and myotube hypertrophy. Additionally, we observed increased AMPK with EX-527 treatment in glucose-restricted conditions in comparison with resveratrol conditions, yet the increase was not significantly increased versus DM conditions and no corresponding changes in P70S6K were observed. These data suggest that only SIRT1 inhibition via EX-527 administration was able to moderately increase AMPK activity in both glucose conditions. Therefore, normal SIRT1 activity may be required for adequate AMPK activity to prevent the suppression of P70S6K and the corresponding reductions in myotube size observed in SIRT1 inhibitor conditions. Despite this, the mechanisms responsible for the increased myotube hypertrophy following resveratrol administration at 24 h in GR conditions were not attributable to alterations in energy sensing signalling and/or protein synthetic signalling. Indeed, previous literature has suggested that SIRT1 activation in myoblasts has been shown to inhibit Akt and leucine evoked increases in mTOR [56]. Alternatively, SIRT1 and 2 have also been previously shown to deacetylate mTOR at Thr-389 with acetylation blocking P70S6K activation and, therefore, deacetylation; resulting in phosphorylation of P70S6K [57]. Furthermore, albeit in cardiac muscle, SIRT1 has been shown to deacetylate Akt and PDK which allows binding to phosphatidylinositol 3,4,5-trisphosphate (PIP(3)), its relocation to the membrane where PDK enables Akt phosphorylation upstream of P70S6K [58]. These later studies are in contrast in the present study where we found no effect of manipulating SIRT activation (assessed by phosphorylation) via resveratrol on P70S6K activity. However, the present study did not assess SIRT1’s deacetylation function, and while phosphorylation of SIRT1 is required to enable increases in deacetylase activity [41], this warrants further investigation. The present study extends the work above by including inhibition of SIRT1 phosphorylation by EX-527, which suggests that normal SIRT1 activity was required to enable adequate AMPK signalling.

## Conclusion

We originally hypothesised that resveratrol treatment would improve differentiation and myotube hypertrophy in differentiating myoblasts, and prevent myotube atrophy in mature differentiated myotubes during glucose restriction (GR). Resveratrol treatment did not improve myoblast differentiation in GR conditions; however, improved hypertrophy of mature myotubes when glucose was available via regulation of slow *Mhyc* gene expression and acutely (for 24 h) maintained myotube size when modelling interstitial low glucose levels mimicking in vivo calorie restriction. Repeated administration of resveratrol every 24 h may therefore be advantageous in vitro during glucose restriction; however, this requires investigation in mammalian models to take into account of altered pharmacokinetics and bioavailability in vivo.
